# ICT-Based Individualized Training of Institutionalized Individuals With Dementia. Evaluation of Usability and Trends Toward the Effectiveness of the InCoPE-App

**DOI:** 10.3389/fphys.2022.921105

**Published:** 2022-07-08

**Authors:** Bettina Barisch-Fritz, Jelena Bezold, Andrea Scharpf, Sandra Trautwein, Janina Krell-Roesch, Alexander Woll

**Affiliations:** Institute of Sports and Sports Science, Karlsruhe Institute of Technology, Karlsruhe, Germany

**Keywords:** digitalization and e-health, Alzheimer’s disease, mobile application, physical activity, feasibility

## Abstract

Physical activity interventions can alleviate the course of disease for individuals with dementia (IWD) who have been extraordinarily affected by the COVID-19 pandemic. Information and Communication Technology (ICT) provides new opportunities not only to mitigate negative effects of the pandemic but also to sustainably improve everyday life of IWD in nursing homes. The aim of the present study was to evaluate the ICT-based InCoPE-App, which was used to assess physical and cognitive performance and deliver individualized exercise for IWD, with regard to 1) user experience of nursing assistants, and 2) trends toward the effectiveness of the intervention on physical and cognitive performance of IWD. An 18-week individualized multidomain intervention (2 × 60 min/session) was delivered to an intervention group (IG; *n* = 10, mean age 88.4 ± 5.6, 70% female) by nursing assistants (*n* = 10, mean age 56.1 ± 10.4, 90% female) using the InCoPE-App. A control group (CG; *n* = 3, mean age 87.3 ± 3.5, 100% female) received conventional treatment. User experience was assessed among nursing assistants by different questionnaires, i.e., PSSUQ and ISONORM 9241/110-S for usability, and AttrakDiff2 for pragmatic (PQ), hedonic quality-identity and stimulation (HQI and HQS), and attractiveness (ATT). Trends toward the effectiveness of the intervention were assessed using MMSE (global cognitive function), FICSIT-4 (balance), 6MWT and TUG (mobility), and m30CST (function of lower limbs). Usability of the InCoPE-App was rated as high by nursing assistants (mean ± SD; overall PSSUQ 2.11 ± 0.75; overall ISONORM 9241/110-S 1.90 ± 0.88; ATT 1.86 ± 1.01; PQ 1.79 ± 1.03; HQI 1.8 ± 0.79; and HQS 1.37 ± 0.69). Dropout was high in the total sample (36.7%). Trends toward the effectiveness were observed within IG in nine IWD who showed positive or neutral trends in at least two physical performance outcomes. Seven participants had positive or neutral trends in the FICSIT-4, seven participants in m30CST, and four and seven participants in 6MWT and TUG, respectively. In conclusion, the InCoPE-App has good nursing assistant-rated usability, whereas training effects and intervention adherence were rather low most likely due to COVID-19 restrictions. Single-subject research revealed more positive than negative trends in IG of IWD. Further research is needed to evaluate feasibility, suitability, and effectiveness of the InCoPE-App.

## 1 Introduction

The COVID-19 pandemic had drastic consequences on global physical activity. For example, an analysis of millions of FitBit users showed declines in physical activity of 7%–38% as compared to pre-pandemic worldwide (FitBit staff 2022). In line with these findings, Trabelsi et al. (2021a) conducted an international online survey with 5,056 replies and reported that during home confinement, daily sitting increased by ∼2 h per day. Significantly negative alterations in sleep patterns and physical activity levels were also observed (Trabelsi et al., 2021a). In nursing homes, daily routines had to be changed due to COVID-19-related restrictions. In particular, various activities and treatments were reduced or canceled and thus, many residents were restrained from being physically active (Frahsa et al., 2020). The consequences of reduced physical activities are manifold, reaching from psychological effects due to isolation up to deterioration of several health outcomes (Kohl et al., 2012). In times of COVID-19, the decreased levels of physical activity in combination with poor sleep quality were identified as predictors of mental wellbeing in older adults (Trabelsi et al., 2021b).

With regard to nursing home residents, two aspects must be noted: First, the already high percentage of sedentary time (up to 90%) in long-term care residents (Souto Barreto et al., 2015) appears to have further increased due to COVID-19-induced restrictions. Second, the progression of the disablement process with regard to physical and cognitive performance may have been accelerated by pandemic-related physical inactivity. A meta-analysis conducted by Tak et al. showed that physical activity is the most effective strategy in preventing and slowing down the disablement process (Tak et al., 2013). Being able to maintain activities of daily living is also associated with a reduced degree of care.

Physical activity interventions have increasingly been valued for their potential to contain the progression of several age-related diseases. One example is dementia, where the preservation of cognitive performance through physical activity interventions is supported by several reviews. For example, the systematic review conducted by Brasure et al. (2018) provided evidence of the effectiveness of physical activity for multidomain interventions. Such interventions, which usually comprise physical activity, diet, and cognitive training, were associated with a delay in cognitive decline. With regard to cohort and case–control studies, Iso-Markku et al. (2022) reported that the risk of all-cause dementia, Alzheimer`s disease, and vascular dementia is decreased by virtue of physical activity. Furthermore, positive effects of physical activity on global cognition, executive function, and memory can be found in older adults with mild cognitive impairment or dementia (Groot et al., 2016; Nuzum et al., 2020; Venegas-Sanabria et al., 2021). Blankevoort et al. (2010) observed that physical activity is beneficial in all stages of dementia and may improve physical functioning and daily living activities (Blankevoort et al., 2010). In general, however, the large heterogeneity of study methodologies and rather the low quality of physical activity interventions in several studies impede the comparability (Blankevoort et al., 2010; Suttanon et al., 2010; Bherer et al., 2013; Forbes et al., 2015; Groot et al., 2016).

Up to 50% of nursing home residents suffer from dementia and often experience disease-specific rapid progression (Gaugler et al., 2014). This target group has been strongly affected by the drastic reduction in therapy programs and treatments caused by COVID-19 restrictions. In light of the absence of a medication to cure dementia, physical activity is one of the major non-pharmacological intervention implemented to treat individuals with Dementia (IWD) (Kouloutbani et al., 2019). Thus, promotion of physical activity interventions even when COVID-19 restrictions are in place is highly relevant. There is a considerable need for interventions that are based on or take into account the needs of IWD, and also assist or train nurses on how to promote and deliver a physical activity program. This was concluded by Altmeier et al. (2021) who conducted a photovoice study on physical activity in eight nursing homes in Germany. To this end, Information and Communication Technology (ICT) may have the potential to address the requirements of both IWD and nursing home employees in order to mitigate the negative effects of the pandemic, and also to sustainably modify the everyday life of IWD in nursing homes. Several innovative approaches and interventions utilizing ICT have found their way into gerontology. For example, beneficial clinical effects were found for walking capacity in older adults after exergaming interventions (Corregidor-Sánchez et al., 2020). Furthermore, DVD-mediated exercise interventions were found to improve physical function in older adults (Wójcicki et al., 2015). Even though these forms of intervention are easily accessible (i.e., no use of Internet, license access, or other material needed), they are not applicable for IWD. For this target group, supervised exercise is essential. Ortiz-Alonso et al. (2020) argue that supervision plays a key role in ensuring a safe and effective physical activity intervention for elderly people (Ortiz-Alonso et al., 2020). Their research showed that a simple supervised exercise program resulted in decreased hospitalization-associated disabilities in hospitalized elderly patients as compared to an unsupervised program (Ortiz-Alonso et al., 2020).

In view of the COVID-19 restrictions, the following two main aspects were addressed in the present study where we aimed at developing an ICT-based solution to tackle physical inactivity in nursing homes: The first was to satisfy the different stakeholder groups, e.g., nursing home assistants, nursing home leadership, etc. (Altmeier et al., 2021). The second was to follow a pragmatic and ecological approach and consider the limited resources of health care institutions such as nursing homes (Aubertin-Leheudre and Rolland 2020). To this end, our team developed an ICT-based intervention delivered via a tablet-based mobile application called InCoPE-App (Individualized Cognitive and Physical Exercise). This application supports nursing assistants in 1) assessing the cognitive and physical performance of IWD, and 2) subsequently training IWD by delivering a multidomain individualized exercise program that automatically adjusts to each IWD by taking into account an individual’s current physical and cognitive performance. This novel and innovative technology thus enables nursing assistants to deliver an individualized physical exercise program through an ICT-based approach. This is particularly noteworthy as it has been postulated that individualization can increase the beneficial impact of physical exercise on dementia prevention (Müllers et al., 2019) as well as cognitive and physical performance in older adults (Barisch-Fritz et al., 2022).

In addition, usability considerations are critically important for a successful adoption of the InCoPE-App by nursing assistants. Even though the number of ICT-based applications implemented in healthcare has increased exponentially, publications addressing their usability are still scarce (Maramba et al., 2019). For example, more than 95% of applications available today have not been scientifically tested, and the limited number of controlled trials on ICT-based interventions reported only modest effects (Free et al., 2013). However, a critical step in the development of ICT-based interventions is examining its usability (Zapata et al., 2015). It is particularly important to consider users’ needs for digital health applications which may lead to a greater acceptance and thus efficiency of the intervention (Brown et al., 2013).

The aim of the present study was to evaluate usability and trends toward the effectiveness of an ICT-based multidomain intervention delivered by the InCoPE-App. Two hypotheses were tested: 1) the InCoPE-App would have a high usability and would be applicable to everyday use by nursing assistants; 2) there would be positive trends toward the effectiveness of the multidomain exercise program imbedded in the InCoPE-App with regard to improving or maintaining the physical and cognitive performance of IWD after an 18-week intervention. To the best of our knowledge, there is no scientifically evaluated application available today that enables nursing assistants who usually lack expertise in sports science to deliver an individualized exercise program to IWD.

## 2 Materials and Methods

### 2.1 Study Design and Sample

The study has been approved by the Ethics Committee of the Karlsruhe Institute of Technology (Germany) and is registered in the German Register of Clinical Trials (drks.de. Identifier: DRKS00024069). The evaluation of the usability (primary outcome) and trends toward the effectiveness (secondary outcome) of the InCoPE-App is based on a field intervention with a cluster-randomized controlled trial. Blinding in terms of group allocation was not applicable for IWD, nursing assistants, or study personnel. The intervention group (IG) received an 18-week ICT-based intervention (for more details, refer to Section 2.2) along with conventional treatment such as individualized medication, standard care, or therapeutic applications.

The ICT-based intervention was delivered by nursing assistants *via* the InCoPE-App. Testing of physical performance of IWD was repeated every 3 weeks. Pre- and post-intervention assessments were conducted for global cognition. Participants of the control group (CG) received their conventional treatment and, similar to participants of the IG, underwent tests to assess physical performance every 3 weeks.

Two different study samples were recruited to evaluate the usability as well as the effectiveness of the InCoPE-App. The first sample (for usability purposes) consisted of nursing assistants which are considered the primary users of the InCoPE-App. This sample was recruited with the assistance of the heads of nursing homes in Southwestern Germany who suggested potentially interested nursing assistants. Inclusion criteria were as follows: 1) willingness to participate in the preparatory training with the InCoPE-App, 2) a high level of fluency in German language, and 3) employment at the nursing home for at least 18 weeks or more. Employees who were externally hired (e.g., physical therapists) were excluded from the study. All nursing assistants received detailed information about the study design and confirmed their willingness to participate by signing an informed consent letter.

The second sample (for effectiveness purposes) included individuals with primary dementia living in nursing homes. Participants of this sample were recommended by nursing assistants based on the following inclusion criteria: 1) Alzheimer’s disease, vascular dementia, or other primary types of dementia; 2) mild-to-moderate stage of dementia (Mini-mental State Examination, MMSE: 10–24); 3) aged 65 years or older; and 4) walking ability of at least 10 m with or without walking aid. Exclusion criteria were as follows: 1) secondary dementia, 2) other severe cognitive impairments, 3) other severe neurological conditions, 4) other severe acute diseases, and 5) severe motor impairments. Prior to the study, written informed consent of participants or their legal guardians was obtained. Prior to the intervention, a medical clearance certificate, containing information about the dementia diagnosis and other pertinent information such as medication intake and comorbidities, was obtained from a participant’s general practitioner.

### 2.2 InCoPE-App and Intervention

The 18-week intervention was delivered *via* the tablet-based (Android) InCoPE-App, which was developed by applying a user-centered approach on the base of earlier work of this research group (Barisch-Fritz et al., 2020). Briefly, the main algorithm implemented in the InCoPE-App to individualize cognitive and physical tasks is based on an unsupervised machine learning approach basically calculated through a cluster analysis of 230 IWD (Barisch-Fritz et al., 2019). The sample was drawn out from IWDs who participated in a previous RCT (Trautwein et al., 2017). The results of the cluster analysis identified four homogeneous subgroups: participants with 1) below-average physical and cognitive performance, 2) average cognitive and above-average physical performance, 3) above-average cognitive and below-average physical performance, and 4) above-average cognitive and physical performance. The algorithm of the InCoPE-App enables the allocation of each IWD based on their cognitive and physical performance data (which are also assessed through the InCoPE-App) to one of these four clusters. The approach contains the calculation of probabilities of cluster affiliation through multinomial logistic regression analysis. The individualized composition of exercises is based on about 700 described exercises which are categorized by means of the primary aim and intensity level of each exercise. The training routine for each individual is generated by the InCoPE-App through cluster allocation for the subsequent 3 weeks.

The contents and functions of the InCoPE-App can be summarized as follows: The first function is to assess cognitive and physical performance of IWD. The second is to design, guide, and monitor an individualized program by combining cognitive and physical exercises depending on an IWD’s current physical and cognitive performance level. These automated functions embedded in the InCoPE-App enable nursing assistants who lack advanced knowledge in sports science and/or psychology to test and individually train IWD. The InCoPE-App contains detailed visual materials and annotations to offer visual support during the execution of the proposed tests and exercises. Prior to the start of the ICT-based intervention, nursing assistants were trained in the correct use of the InCoPE-App. This training comprised online modules for self-study (e.g., theoretical basics of adapted physical activity with IWD, objective testing of physical and cognitive performance in IWD, and first steps with the InCoPE-App) and two face-to-face training sessions on the use of the InCoPE-App.

The tests assessing physical and cognitive performance included in the InCoPE-App are based on recommendations from previous studies and were previously administered to IWD (Bossers et al., 2016; Trautwein et al., 2019a) (refer to Section 2.4 for details). The initial testing of physical and cognitive performance is critically important in order to assign the IWD to one of the four training clusters as described above. Physical performance assessments were repeated every 3 weeks during the 18-week intervention period to allow the adjustment of duration, intensity, and contents of physical and cognitive exercises *via* InCoPE-App. We anticipate that the desired physical and cognitive adaptation to the exercise program will be achieved by gradually increasing exercise difficulty and intensity (e.g., number of repetitions).

One-to-one training sessions between nursing assistant and each IG participant were conducted twice a week. Each 60-min session comprised of the following: 1) a ritualized warm-up, 2) a first set of exercises, 3) a short break with ritualized balance exercises, 4) a second combination of exercises, and 5) a cool down at the end. Actual training duration was about 40 min. The ritualization of training sequences is important to give IWD a sense of security and familiarity (Schick 2015). The warm-up and cool down included walking exercises for the promotion of endurance as well as general mobilization tasks. The main training program took place in the first and second set of exercises. Within these two sets of exercises, strength, coordination, and balance were addressed. Most exercises were combined with a cognitive task (e.g., counting repetitions while doing strength exercises or recall previously learned commands while playing ball). Exercise composition was defined based on the cluster allocation of each individual. For example, an IWD in cluster 1 received exercises focusing on balance, mobility, and strength, as well as cognitive stimulation.

### 2.3 Measurements

#### 2.3.1 Evaluation of Usability and Feasibility

The primary outcomes, i.e., usability and feasibility of the InCoPE-App, were investigated using the Post-Study System Usability Questionnaire (PSSUQ) and the ISONORM 177 9241/110-S questionnaire. Perceived hedonic and pragmatic quality was assessed by AttrakDiff2 questionnaire (see Table 1). The feasibility outcomes were defined by the logging events.

**TABLE 1:** Primary outcomes for usability and feasibility of the InCoPE-App.

Dimensions	Assessments	Pre	Post	Implemented in the InCoPE-App
Overall usability	Post-study system usability questionnaire, PSSUQ version 3 Lewis (2002)		•	No
Overall usability	Questionnaire operationalizes the seven criteria of the DIN EN ISO 9241-110; ISONORM 9241/110-S Prümper (1997)		•	No
Perceived hedonic and pragmatic quality	AttrakDiff2 (Hassenzahl et al. (2003)		•	No
Logging events for feasibility	Number of completed training sessions	During the intervention		Yes
Logging events for feasibility	Number of completed assessments	During the intervention		Yes

The overall usability was assessed after the 18-week intervention, i.e., nursing assistants who had used the InCoPE-App to deliver the multidomain intervention to IWD completed the three questionnaires as paper–pencil assessments. The PSSUQ version three was used to obtain an insight into user satisfaction with the system, e.g., system usefulness, information quality, and interface quality (Lewis 2002). This questionnaire, which is considered a useful tool for field studies, comprises 19 items rated on a 7-point Likert scale (1–strongly agree to 7–strongly disagree) (Lewis 2002; Sauro and Lewis 2012). The PSSUQ is interpreted through the calculated average score (OVERALL), system usefulness (SYSUSE), information quality (INFOQUAL), and interface quality (INTERQUAL). To assess the calculated scores, reference values from Sauro and Lewis (2016) were used (Sauro and Lewis 2016).

The International Organization for Standardization (ISO) suggested seven criteria based on the International Ergonomics Standard DIN EN ISO 9241-110 which have to be considered when developing ICT. The ISONORM 9241/110-S questionnaire covers the seven defined criteria and has been developed to evaluate software development (Prümper 1997). The processing takes 10 min during which bipolar statements from 21 items need to be rated on a 7-point Likert scale (1–very negative to 7–very positive) (Prümper 1997). The 21 items cover seven interaction principles: task suitability (TASKSUIT), self-descriptiveness (SELFDE), expectation conformity (EXPECTCON), learnability (LEARN), controllability (CONTROL), robustness (ROBUST), and user engagement (USEREN). The value of 1 indicates a minimum criterion for “good” software. Smaller values indicate areas for improvement (Prümper 1997).

The perceived hedonic and pragmatic quality of the InCoPE-App was assessed by the AttrakDiff2 questionnaire (Hassenzahl et al., 2003). This holistic evaluation of the user experience examines the participants’ perception of the InCoPE-App by means of semantic differentials along 28 items. The items are coded from −3 to 3 with zero being the neutrality of the answers. The four perspectives are pragmatic quality (PQ), hedonic quality (divided into identity (HQI) and stimulation (HQS), and attractiveness (ATT) (Hassenzahl et al., 2003).

The logging events were recorded within the InCoPE-App during the entire 18-week intervention to obtain deeper insights into feasibility. The feasibility measures were calculated to estimate practicality and acceptance of the App-based individualized exercise program and assessments. To this end, the analysis of logging events was used to determine the frequency of completing training sessions, as well as assessment sessions.

#### 2.3.2 Evaluation of Trends Toward the Effectiveness on Physical and Cognitive Performance

Secondary outcomes were assessed to examine the effectiveness of the 18-week ICT-based intervention as delivered by nursing assistants. The assessments focused on physical and cognitive performance (see Table 2). We tested global cognition using the Mini-mental State Examination Test [MMSE (Folstein et al., 1975)], balance using the Frailty and Injuries: Cooperative Studies of Intervention Techniques (FICSIT-4) (Rossiter-Fornoff et al., 1995), function and strength of the lower limbs using the Modified 30-s Chair–Stand Test (CST) (Jones et al., 1999), and mobility using the 6-meter Walking Test (6MWT) (Graham et al., 2008) and Timed Up & Go test (TUG) (Podsiadlo and Richardson 1991). All test results were recorded and entered into the InCoPE-App.

**TABLE 2:** Secondary outcomes for the effectiveness of the InCoPE-App.

Dimensions	Assessments	Pre	Post	Implemented in the InCoPE-App
Global cognition	Mini-mental state examination Test, MMSE Folstein et al. (1975)	•	•	Yes
Mobility	Timed up & Go test, TUG Podsiadlo and Richardson (1991)	•	•	Yes
Mobility	Six-meter walking test, 6MWT Graham et al. (2008)	•	•	Yes
Function and strength of lower limbs	Modified 30-s chair–stand test, m30CST Jones et al. (1999)	•	•	Yes
Balance	Frailty and injuries: cooperative studies of intervention techniques–subtest 4, FICSIT-4 Rossiter-Fornoff et al. (1995)	•	•	Yes
Sociodemographic data and medical information	Age, sex, dementia form and severity, depression severity, and Cumulative illness rating scale–severity index, CIRS Linn et al. (1968)	•		No

The MMSE (Folstein et al., 1975) was implemented in the InCoPE-App. The MMSE assesses seven cognitive domains, e.g., working memory, visuospatial skills, and language. The test–retest reliability of the MMSE was previously confirmed by Folstein et al. (1975) and Tombaugh and McIntyre (1992), as the inter-class correlation coefficient (ICC) was higher than 0.8 in both studies (Folstein et al., 1975; Tombaugh and McIntyre 1992).

Static balance was determined using FICSIT-4 (Rossiter-Fornoff et al., 1995). Participants were asked to perform four different standing positions (i.e., Romberg, semi-tandem, tandem, and single leg) for 10 seconds without walking aids or any other kind of assistance. The FICSIT-4 performance was rated on a scale of 0–5 points. If participants could not hold the first position for at least 3 s, a score of 0 was given. In contrast, participants received a score of 5 if they were able to stand in the most difficult, i.e., single-leg position, for at least 10 s.

The function and strength of lower limbs were assessed using the m30CST. Participants were asked to stand up from a chair (46 cm high, with armrests) as many times as possible within 30 s. The modified version allows the use of armrests (Applebaum et al., 2017), which is essential for the majority of older IWD to safely perform this test. No walking aids were allowed. The higher the number of repetitions the better the function and strength of lower limbs.

The 6MWT (Graham et al., 2008) was performed to assess mobility. Participants were asked to walk a marked distance of 6 m as fast as possible. The InCoPE-App recorded the time each participant took to cover the 6-m distance. In front of the starting line and behind the finish line, participants had a space of about 2 m for acceleration and deceleration. If needed, participants were allowed to use their walking aids. The shorter the required time, the better the mobility performance.

The TUG as second test for assessing mobility was also administered using the InCoPE-App. Nursing assistants asked participants to stand up from a chair, walk 3 m, then turn around and walk back to sit on the chair again (Podsiadlo and Richardson 1991). The time elapsed between start and completion of the test pattern was recorded by the InCoPE-App. Time was measured from the initial impulse to stand up until participants were seated again. Everyday walking aids were allowed. The shorter the required time, the better the mobility performance.

The chosen tests for physical performance are considered reliable tests in a geriatric setting. When administered to IWD, test–retest reliability was ICC = 0.79–0.82 for FICSIT-4, ICC = 0.83–0.89 for 6MWT, ICC = 0.78–0.88 for m30CST, and ICC = 0.72–0.99 for TUG (Blankevoort et al., 2013; Trautwein et al., 2019b). Minimal Detectable Change _95%_ is 58.9–71.1% for FICSIT-4, 31.6–41.5% for 6MWT, 33.2–45.7% for m30CST, and 15.8–39.6% for TUG (Blankevoort et al., 2013; Trautwein et al., 2019b). No information about content and construct validity of the tests are available in this setting.

Sociodemographic data (e.g., age and sex) and pertinent medical information (e.g., dementia type and severity, presence of depression, and Cumulative Illness Rating Scale (CIRS) (Linn et al., 1968)) were obtained from nursing assistants or general practitioners before the start of the intervention *via* paper–pencil forms.

### 2.4 Statistical Analysis

With regard to the examination of the usability of the InCoPE-App, we conducted descriptive analyses. Results from PSSUQ, ISONORM 9241/110-S, and AttrakDiff2 are presented in means, standard deviations (SD), and 95% confidence intervals and compared to published reference values. Logging events for feasibility are also descriptively presented with cumulative numbers and percentages as well as means of adherence to the IG.

The trends toward the effectiveness of the ICT-based individualized intervention were descriptively described using means ± SD. The differences within the groups were tested using paired sample t-test to compare physical and cognitive performance at pre- and post-assessments. To get a deeper insight into the trends toward the effectiveness, a single-subject research design was applied. We conducted a linear regression analysis over the seven assessment times of the four physical performance variables (Johnson and Ottenbacher 1991). The results are reported as regression coefficients (*β*), the standard error of estimates (SE), and the coefficient of determination (R^2^). The trends for each individual were coded based on the regression coefficient. A positive trend (coded with +) was set at *β* ≥ 0.1 for FICSIT-4 and m30CST, as well as *β* ≤ -0.1 for 6MWT and TUG. No trend (coded with 0) was set at −0.1< *β* > 0.1. A negative trend (coded with −) was set for *β* ≤ −0.1 for FICSIT-4 and m30CST, as well as *β* ≥ 0.1 for 6MWT and TUG.

## 3 Results

### 3.1 Results Regarding Usability and Feasibility

#### 3.1.1 Usability Scales

Results from 10 nursing assistants (mean age 56.1 ± 10.4 years, 90% female) for the three different usability scales collected after the 18 weeks ICT-based intervention are presented in Table 3. The overall PSSUQ was rated with a mean of 2.11 ± 0.75, and the overall ISONORM 9241/110-S with a mean of 1.90 ± 0.88. The four perspectives of the perceived hedonic and pragmatic quality of the InCoPE-App were rated with PQ 1.79 ± 1.03, HQI 1.81 ± 0.79, HQS 1.37 ± 0.69, and ATT 1.86 ± 1.01.

**TABLE 3:** Results on usability based on PSSUQ, ISONORM 9241/110-S, and AttrakDiff2.

Dimension	Mean	± SD	Confidence interval	Mean range of items	References
PSSUQ					Sauro and Lewis (2016)
SYSUSE	1.99	±0.75	1.44–2.54	1.00–3.00	2.80
INFOQUAL	2.31	±0.82	1.73–2.90	1.00–3.57	3.02
INTERQUAL	2.00	±0.70	1.50–2.50	1.00–3.25	2.49
Overall	2.11	±0.75	1.57–2.65	1.00–3.26	2.82
ISONORM 9241/110-S					Prümper (1997)
TASKSUIT	1.80	±0.89	1.16–2.44	0.00–3.00	mean values = 1: practical benchmark; minimum criteria for “good software”; mean values <1: optimization needs
SELFDES	1.80	±1.05	1.05–2.55	0.00–3.00	
EXPECTCON	1.97	±0.96	1.28–2.66	0.00–3.00	
LEARN	2.00	±0.99	1.29–2.71	0.00–3.00	
CONTROL	1.60	±1.02	0.87–2.33	0.00–3.00	
ROBUST	1.90	±1.11	1.10–2.70	0.00–3.00	
USEREN	2.23	±1.01	1.51–2.95	0.00–3.00	
Overall	1.90	±0.88	1.27–2.53	0.00–3.00	
AttrakDiff2					No References values
PQ	1.79	±1.03	1.15–2.42	0.70–2.20	
HQI	1.81	±0.79	1.33–2.30	1.50–2.50	Comparison with the highest value of 3
HQS	1.37	±0.69	0.95–1.80	0.30–2.00	
ATT	1.86	±1.01	1.23–2.48	1.70–2.10	

PSSUQ, Post-Study System Usability Questionnaire; SYSUSE, system usefulness; INFOQUAL, information quality; INTERQUAL, interface quality; ISONORM, 9241/110-S = questionnaire operationalizes the seven criteria of the DIN EN ISO, 9241-110; TASKSUIT, task suitability; SELFDE, self-descriptiveness; EXPECTCON, expectation conformity; LEARN, learnability; CONTROL, controllability; ROBUST, robustness; USEREN, user engagement; AttrakDiff2 = Questionnaire for perceived hedonic and pragmatic quality; PQ, pragmatic quality; HQI, hedonic quality identity; HQS, hedonic quality stimulation; ATT, attractiveness.

#### 3.1.2 Logging Events for Feasibility

**FIGURE1:**
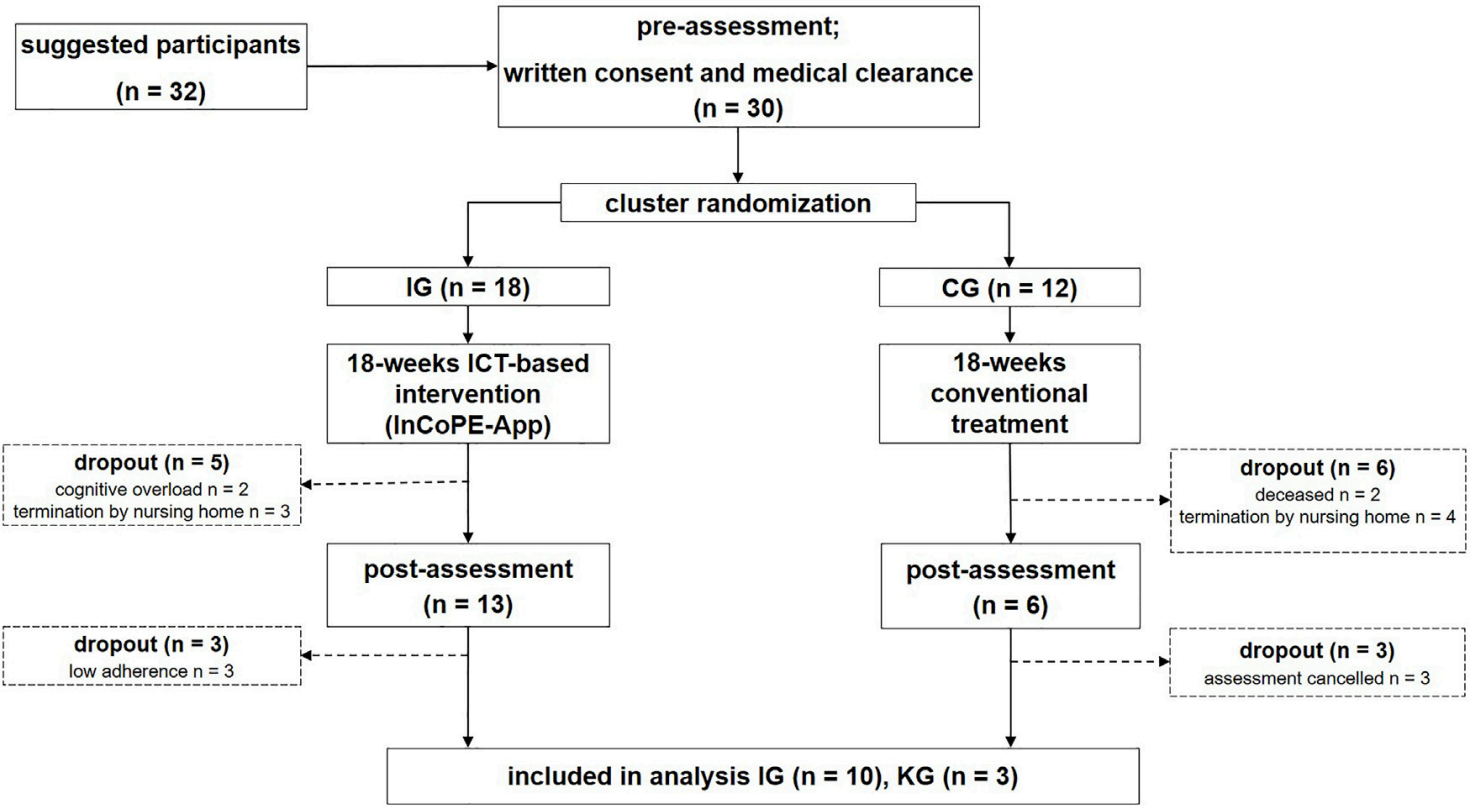
Flow of participants.

Results of the logging events are presented in Table 4. The pre-assessments for the inclusion of the participants were completed by 18 participants of the IG and 12 participants of the CG. The post-assessments were completed by 13 participants of the IG and six participants of the CG which represents 72.2% and 50.0%, respectively (see Figure 1, Section 3.2.1).

**TABLE 4:** Results on feasibility based on logging events.

	Completed assessments		Completed training sessions			Adherence mean ± SD	
	Pre (included participants)	Post	>25%	>50%	>75%	Total (n=18)	>25% (n=10)
IG n (%)	18 (100%)	13 72.2%	14	9	7	18	10
			78.6%	64.3%	50%	52.3% ± 41.8	85.3% ± 20.5
CG n (%)	12 (100%)	6 50.0%	n.a.	n.a.	n.a.	n.a.	n.a.
			n.a.	n.a.	n.a.	n.a.	n.a.

IG, intervention group; CG, control group; SD, standard deviation.

Participants of the IG had a total mean adherence of 52.3% ± 41.8%. Among participants who attended more than 25% of the total 36 training sessions, the mean adherence was 85.3% ± 20.6%. An adherence of 75% or more was achieved by 50% of the participants.

### 3.2 Results Regarding Effectiveness on Physical and Cognitive Performance

#### 3.2.1 Sample Characteristics

Ten nursing assistants delivered the ICT-based intervention with the InCoPE-App. The pre-assessments were performed with 30 IWD participants, and post-assessments with 19 IWD participants of which 13 participants could be included in the final analysis (see Figure 1). The dropout rate from pre- to post-intervention assessments for the total sample was 36.7% and 27.8% for the IG, respectively. The reasons for the dropouts were death (*n* = 2), cognitive overload (*n* = 2), and termination of the intervention by responsible nursing assistants (*n* = 7).

The sample characteristics of the 13 included participants are presented in Table 5. The mean age of the total sample was 88.2 years ±5.1, and 77% of the participants were females. Dementia diagnosis was present in 77% of the total sample with 31% having Alzheimer’s disease, 31% vascular dementia, 8% dementia of mixed etiology, and 31% having a non-determined type of dementia. One participant also had a diagnosis of major depression. The mean CIRS severity index was 1.29 ± 0.30.

**TABLE 5:** Sample characteristics of IWD.

Sample	Age (years)	Sex	Dementia diagnosis^a^	Dementia form^a^	Dementia severity^a^	Depression diagnosis^a^	CIRS Severity Index
	Mean ± SD	n (%)	n (%)	n (%)	n (%)	n (%)	Mean ± SD
IG (*n* = 10)	88.4 ± 5.6	7 (70%) female	7 (70%) present, 1 (10%) no, 2 (20%) unknown	4 (40%) AD, 2 (20%) vascular, 1 (10%) mixed, 3 (30%) unknown	4 (40%) mild, 3 (30%) moderate, 3 (30%) unknown	1 (10%) present, 8 (80%) no, 1 (10%) unknown	1.33 ± 0.38
CG (*n* = 3)	87.3 ± 3.5	3 (100%) female	3 (100%) present	2 (67%) vascular, 1 (33%) unknown	3 (100%) moderate	2 (67%) no	1.24 ± 0.21
Total (*n* = 13)	88.2 ± 5.1	10 (77%) female	10 (77%) present, 1 (8%) no, 2 (15%) unknown	4 (31%) Alzheimer, 4 (31%) vascular, 1 (8%) mixed, 4 (31%) unknown	4 (31%) mild, 6 (46%) moderate, 3 (23%) unknown	1 (8%) present, 10 (77%) no, 2 (15%) unknown	1.29 ± 0.30

IG, intervention group; CG, control group, n, number, SD, standard deviation; CIRS, cumulative illness rating scale; AD, Alzheimer`s disease

a Information gathered through the general practitioner of the IWD., Additional information on dementia severity can be found in Table 6 based on the results of the MMSE.

#### 3.2.2 Comparison of Pre- and Post-Intervention Assessments

Results on the cognitive and physical performance variables are presented in Table 6. The comparison of the pre- and post-intervention assessments was statistically significant for FICSIT-4 (balance) in the CG (pre: 2.00 ± 0.50, post: 3.33 ± 0.58). All other findings were not statistically significant when comparing means between pre- and post-intervention assessments.

**TABLE 6:** Results on the effectiveness.

	MMSE			FICSIT-4			6MWT			TUG			m30CST		
	Pre	Post	Time effect	Pre	Post	Time effect	Pre	Post	Time effect	Pre	Post	Time effect	Pre	Post	Time effect
	Mean ± SD	Mean ± SD	t (df), p	Mean ± SD	Mean ± SD	t (df), p	Mean ± SD	Mean ± SD	t (df), p	Mean ± SD	Mean ± SD	t (df), p	Mean ± SD	Mean ± SD	t (df), p
IG (*n* = 10)	18.60 ±2.88	19.30 ±4.62	t (9) = 5.546, *p* = 0.599	2.80 ±1.03	3.55 ±1.32	t (9) = 1.366, *p* = 0.205	10.75 ±4.53	11.63 ±8.60	t (9) = 0.240, *p* = 0.817	19.30 ±9.90	18.40 ±6.42	t (9) = 0.401, *p* = 0.698	8.0 ±3.7	9.1 ±2.3	t (9) = 1.160, *p* = 0.276
CG (*n* = 3)	18.00 ±3.51	19.00 ±5.57	t (2) = 0.655, *p* = 0.580	2.00 ±0.50	3.33 ±0.58	t (2) = 8.000, *p* = 0.015	10.67 ±2.52	10.33 ±2.52	t (2) = 1.000, *p* = 0.423	22.67 ±9.07	21.67 ±6.51	t (2) = 0.433, *p* = 0.707	7.5 ±2.5	9.0 ±4.2	t (2) = 3.000, *p* = 0.205
Total (*n* = 13)	18.46 ±3.53	19.23 ±4.60	t (12) = 0.754, *p* = 0.465	2.62 ±1.02	3.50 ±1.17	t (12) = 2.085, *p* = 0.059	10.73 ±3.95	11.27 ±7.31	t (12) = 0.209, *p* = 0.839	20.08 ±7.18	19.15 ±6.32	t (12) = 0.524, *p* = 0.610	7.9 ±3.5	9.1 ±2.4	t (12) = 1.483, *p* = 0.166

IG, intervention group; CG, control group; SD, standard deviation; MMSE, Mini-mental State Examination Test; FICSIT-4, Frailty and Injuries: Cooperative Studies of Intervention Techniques-subtest 4, 6MWT, 6-meter Walking Test; TUG, Timed Up & Go test, m30CST, modified 30 second Chair-Stand Test, df, degree of freedom.

#### 3.2.3 Trends Toward the Effectiveness by Applying Single-Subject Analysis

**FIGURE 2:**
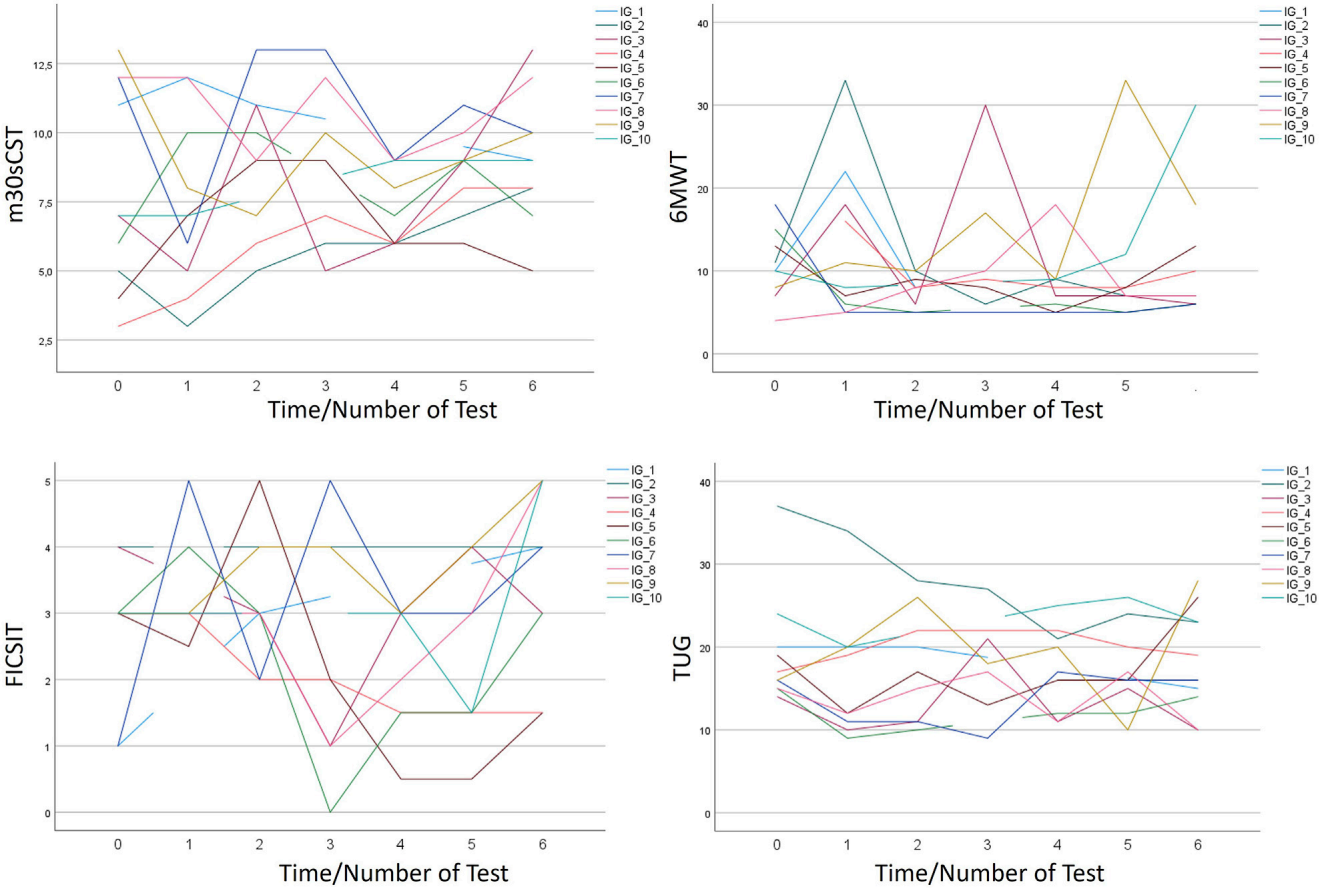
Individual development of physical performance based on 6MWT, FICSIT-4, m30CST, and TUG tests.

Figure 2 shows the trends toward the effectiveness of the ICT-based intervention within the IG and over the seven assessment times. A decrease in time needed to complete the 6MWT and TUG tests indicates a positive trend, whereas an increase in numbers of m30CST and FICSIT-4 scores indicates a positive trend.

Nine participants showed positive or neutral trends in two or more variables assessing physical performance (see Table 7). One participant showed only positive trends for balance. Positive and neutral trends were found in seven participants for FICSIT-4, in seven participants for m30CST, in seven participants for 6MWT, and in four participants for TUG.

**TABLE 7:** Trends toward the effectiveness.

Individual	FICSIT-4			6MWT			TUG			m30CST			Individual trend (FICSIT-4, 6MWT, TUG, m30CST)
	β	SE	R^2^	β	SE	R^2^	β	SE	R^2^	β	SE	R^2^	
IG-1	0.464	0.802	0.862	−1.000	10.614	0.017	−0.904	0.951	0.904	−0.422	0.728	0.777	+, +, +, −
IG-2	0.000	0.000	—	−2.321	8.831	0.279	−2.464	2.735	0.820	0.643	0.878	0.750	0, +, +, +
IG-3	−0.043	1.220	0.007	−0.857	9.850	0.041	-0.071	4.352	0.002	0.750	2.907	0.272	0, +, 0, +
IG-4	−0.286	0.293	0.842	−0.886	2.962	0.281	0.286	2.028	0.100	0.821	0.788	0.859	−, +, −, +
IG-5	−0.464	1.328	0.406	−0.071	3.282	0.003	1.000	4.472	0.219	−0.071	2.077	0.007	−, 0, −, 0
IG-6	−0.232	0.912	0.312	−1.000	3.422	0.374	0.179	2.505	0.034	−0.071	1.916	0.010	−, +, −, 0
IG-7	0.214	1.558	0.096	−1.286	4.375	0.326	0.571	3.295	0.144	0.000	2.746	0.000	+, +, −, 0
IG-8	0.179	1.262	0.101	0.821	4.708	0.146	−0.321	3.032	0.059	−0.143	1.568	0.044	+, −, +, -
IG-9	0.250	0.579	0.510	2.607	7.383	0.411	0.357	6.570	0.016	−0.214	2.104	0.055	+, −, −, −
IG-10	0.108	1.401	0.051	2.358	7.911	0.443	0.388	2.392	0.190	0.403	0.386	0.907	+, −, −, +
Total IG mean	−0.001	0.492	0.000	−0.094	2.119	0.011	−0.071	0.093	0.032	0.149	0.746	0.182	
Variable trend	+ = 5; 0 = 2; − = 3			+ = 5; 0 = 1; − = 4			+ = 3; 0 = 1; − = 6			+ = 4; 0 = 3; − = 3			

SE, standard error of estimates; *β*, regression coefficient; R2, coefficient of determination; FICSIT-4, Frailty and Injuries: Cooperative Studies of Intervention Techniques–subtest 4, 6MWT, 6-m Walking Test; TUG, Timed Up & Go test, m30CST, modified 30-s Chair–Stand Test, +, positive trend (*β* ≥ 0.1 for FICSIT-4, and m30CST, *β* ≤ −0.1 for 6MWT, and TUG), 0, no trend (−0.1< *β* > 0.1), and −, negative trend (*β* ≤ −0.1 for FICSIT-4, and m30CST, *β* ≥ 0.1 for 6MWT, and TUG).

## 4 Discussion

The InCoPE-App can be used by nursing assistants to deliver an ICT-based multidomain intervention to individually train IWD in a nursing home setting. To the best of our knowledge, this is the first study that evaluates the usability, feasibility, and trends toward the effectiveness of a novel and innovative ICT-based physical exercise intervention delivered by nursing assistants. The App enables nursing assistants to deliver a scientifically substantiated and individualized exercise program among institutionalized IWD. The findings are discussed along the two hypotheses focusing on 1) usability and feasibility, and 2) trends toward the effectiveness of the InCoPE-App on physical and cognitive performance.

### 4.1 Usability and Feasibility

The first hypothesis addressed the usability and feasibility of the InCoPE-App. The usability was evaluated with three questionnaires focusing on different aspects of user experience. In conclusion, all results were above reference values (which are available for PSSUQ and ISONORM 9241/110-S). Thus, the user experiences of nursing assistants with the InCoPE-App can be regarded as exceptionally satisfactory.

The findings for the PSSUQ can be interpreted by using the means determined by Sauro and Lewis (2016) involving 21 studies and 210 participants (i.e., the lower the score, the better the user experience). The mean values from our study ranged from 1.99 to 2.31 and thus, are better than the means presented by Sauro and Lewis (2016). This instrument has been shown to be effective in small samples and is also highly sensitive in evaluating user experience (Tullis and Stetson 2004). Thus, the InCoPE-App performs well with regard to “information quality,” “interface quality,” and “system usefulness.”

Findings of the ISONORM 9241/110-S questionnaire that operationalizes the seven criteria of the DIN EN ISO 9241-110 (Prümper 1997) were also rated as high (overall mean 1.90 ± 0.88). The mean values ranged from 1.60 to 2.23 and are thus all above the practical benchmark which serves as minimum criterion for a good software. The seven criteria of the DIN EN ISO 9241-110 are principles of interaction regarding the ergonomics of the human–system interaction. Our findings showed that these principles of interaction are well implemented in the InCoPE-App, and no further improvement or adjustment of the App in this regard is needed.

The findings of the perceived hedonic and pragmatic quality of the InCoPE-App were highest rated for the global attractiveness which indicates that the nursing assistants had good overall experiences with the InCoPE-App. Compared to the highest possible rating of three points, the ATT mean of 1.86 ± 1.01 is rather high. The pragmatic quality was also rated as high (mean 1.79 ± 1.03), thus indicating that the usefulness of the InCoPE-App as well as embedded functions were perceived as valuable and applicable in nursing assistants’ daily routines. These findings are in line with the category “system usefulness” of the PSSUQ, which was also highly rated. The values for identity (mean 1.81 ± 0.79) of the perspective hedonic quality were also rated as high. Thus, nursing assistants may have established a closer relationship with the trained IWD through using the InCoPE-App. This might be valuable and can be regarded as a desired working condition, particularly in light of rather precarious working situations in nursing homes (Kliner et al., 2017). The values of the second subdimension, i.e., hedonic quality stimulation, are rated lower (mean 1.37 ± 0.69) compared to the other dimensions. This perspective implies the need for improving own knowledge and proficiency (Hassenzahl et al., 2003). The lower rating on this scale may imply that the nursing assistants felt that they did not experience self-improvement in the field of physical activity promotion or instruction. Further examination of this perceived lack of self-improvement is needed. The rating for the perceived hedonic and pragmatic quality and attractiveness were high compared to other studies using this instrument (Trahms et al., 2017; Mayer et al., 2019; Müller et al., 2021). However, the direct comparison of these findings to previous observations is difficult due to the uniqueness of the InCoPE-App.

In general, the nursing assistants, i.e., users, regarded the InCoPE-App as useful and attractive with a well-designed interaction between human being and technology. Furthermore, they experienced the interaction with the residents guided by the InCoPE-App as valuable. These positive results in user experience did not align with our results with regard to the feasibility. The analyzed logging events focusing on the completed training sessions and physical and cognitive performance assessments of IWD were rather low. Of 30 included IWD, only 50% of the CG and 72% of the IG performed the post-intervention assessments. The overall dropout rate of 36.7% from pre- to post-intervention assessment is very high compared to other studies that conducted physical exercise interventions (Eggermont et al., 2006; Puente-González et al., 2021). Multidomain supervised interventions are generally regarded as feasible and safe with low dropout rates (Park et al., 2019). However, in samples of institutionalized IWD, the dropout rates are often higher (Eggermont et al., 2006). For example, Puente-González et al. (2021) had a dropout rate after 3 months of 19% and after 6 months of 32%. They conducted a multidomain exercise program for IWD delivered in a group setting. In our study, one potential explanation for the relatively high drop-out rate may be the COVID-19 pandemic and related restrictions. Our study was planned and conducted during and between two long periods of lockdowns, in which especially nursing homes were severely affected by contact restrictions. Two nursing homes terminated their participation in our study due to a high number of COVID-19 cases among employees and residents. However, one aim of the ICT-based intervention was to make nursing homes independent from outside exercise and fitness experts; thus, we developed an ICT-based app to enable nursing assistants to test and train residents individually. This approach would also allow them to comply with hygiene and safety measures related to COVID-19. In light of our results, this aim may not have been sufficiently achieved. We can hypothesize that not the possibility to deliver the ICT-based intervention during the lockdowns presented a problem to nursing assistants, but rather the already precarious employment conditions in this setting which were further exacerbated by additional workload and work absences during the pandemic (Wang et al., 2020; Veronese and Barbagallo 2021). However, this needs to be further examined and additional adherence support strategies such as from van der Wardt et al. (2017) need to be considered.

In contrast, the overall adherence of 52.3% in our study may appear lower compared to previously reported mean adherence of 70% in IWD and individuals with mild cognitive impairment (Di Lorito et al., 2020). However, considering the reasons for dropout in our research (e.g., termination of the intervention by the nursing home itself or illness), the remaining sample that participated in more than 25% of the training sessions had an adherence of 85.3%. This means that, on average, the participants fulfilled 30 of 36 possible training sessions.

### 4.2 Trends Toward the Effectiveness of Physical and Cognitive Performance

The findings on the effectiveness of the ICT-based intervention on physical and cognitive performance were not statistically significant when comparing the pre- and post-intervention assessments. The only statistically significant finding was an improvement of balance in the CG. The CG continued their conventional treatment during intervention phase which was not further documented. However, it is possible that this conventional treatment also contained movement-related treatments like physiotherapy or fall prevention, where balance performance may have been addressed. Thus, these findings may be biased. More importantly, the sample size of CG was very small and cannot be adequately interpreted based on inferential statistics. In addition, further studies in this setting should focus on the documentation of the conventional treatment.

With regard to the second hypothesis that postulated positive trends after the 18-week ICT-based intervention, we examined the findings on physical performance by using a single-subject research design over the treatment phase. The fitting of trendlines was used before to objectify courses of changes in single subjects (Johnson and Ottenbacher 1991; Zhan and Ottenbacher 2001). This procedure was chosen as the sample size in our study was too low for applying inferential statistics. The descriptive comparison of the means from pre- to post-intervention assessments revealed that there is a positive shift in the mean cognitive and physical performance (except for 6MWT) in not only the IG but also the CG. Physical performance of the CG was also tested every 3 weeks while receiving conventional treatment. It can be speculated that there was a learning effect that could also have been overlaid by additional conventional treatment.

The analysis of single subjects of the IG over the seven assessment times showed more positive than negative trends. The approach to insert a trendline based on linear regression helps to assess and quantify the trend (Johnson and Ottenbacher 1991). However, the fit of the trendline is not in all cases acceptable regarding the small R^2^ values. This is not surprising considering the variation in the intra-subject measurement results. The variations indicate that there might be several disturbing influences, which is in line with the partly low absolute reliability of the physical performance tests in the sample of IWD (Trautwein et al., 2019b). One potential explanation may be that some assessment tools were not specifically developed for IWD and thus might be impacted by the cognitive disorder (Trautwein et al., 2019b). Another explanation might be that strong daily fluctuations, e.g., with regard to mood or motivation, exist in this target group (Cipriani et al., 2020). Thus, further research and different analysis approaches, for example, on the base of exponential smoothing, are needed to take daily fluctuations in IWD into account.

### 4.3 Limitations

The main limitation of this study was the small sample size to examine the effectiveness of the ICT-based intervention. The main reason for the small sample is that the study was conducted during the challenging time of the COVID-19 pandemic. Furthermore, the drastically reduced number of IWD in the CG did not allow for a direct comparison of the IG and CG. Thus, our results should be considered preliminary and need to be verified through a randomized controlled trial with a sufficient number of participants as calculated by a power analysis. Furthermore, a following study should integrate information on the amount and content of the conventional treatment for both the IG and the CG.

Another limitation pertains to the assessment tools used for the sample of IWD. This might explain the high intra-subject variation for the results on the physical performance. The partly low absolute reliability of these tools should be further analyzed, and specific and meaningful assessment tools for IWD to assess physical performance should be developed (Trautwein et al., 2019b). Further reasons for the intra-subject variation may be daily fluctuations which should also be examined in future studies. For example, we also collected information about the state of daily form (e.g., with regard to hunger, thirst, sleep, etc.) which was assessed before each training session. The analysis of this information might be a first step to understanding the potential impact of daily fluctuations on physical and cognitive performance in IWD. Other potential mediating or moderating variables that may have affected our study findings are, e.g., frailty, fear of falling, physical fitness readiness, or body composition. In particular, nutrition is a relevant factor when considering cognitive performance (Masoomi et al., 2020; Dominguez et al., 2021). We will document these factors in subsequent studies to analyze these random effects by applying linear mixed models and possibly identify variables that are additionally relevant for the individualization of multidomain exercise interventions.

In general, our individualization approach based on a one-to-one training between nursing assistant and IWD can be regarded critically. On the one hand, the suitability of the daily use of the ICT-based InCoPE-app in nursing homes, which often have a shortage of employees, is imminent and must be critically discussed. Further research in the field of structure evaluation is thus needed. On the other hand, multidomain exercise programs delivered in a group setting seem to have additional benefits for cognitive performance (Kelly et al., 2014). This controversy might be solved by a larger study using the InCoPE-app where more IWD will be tested and trained and thus, small groups for each cluster can be formed. Within these similar clusters, the group setting effects may even be higher as all participants have a similar physical and cognitive performance level. In addition to these considerations, the InCoPE-App might be extended in several ways. One option is to modify the app in a way that will increase the interaction between nursing assistants, i.e., users with IWD. Another option is to implement film material as additional content, which may allow for the InCoPE-App to be used in home care, e.g., by family caregivers. A large number of physical exercises can be carried out with little equipment and limited space at low cost as demonstrated by the vast number of YouTube videos promoting physical exercise in a home setting during COVID-19 (Vancini et al., 2022).

## 5 Conclusion

This study reports the usability, feasibility, and trends toward the effectiveness on physical and cognitive performance of an ICT-based intervention, namely the InCoPE-App, and introduces several considerations and approaches for a scientific evaluation of such a technology. In conclusion, the high satisfaction reported among users is positive and confirms a successful user-centered development approach. However, the positive user experience of the nursing assistants was not reflected in the training effects and adherence to the intervention. The main reason for the high drop-out rate may be the difficult conditions within nursing homes during the COVID-19 lockdowns.

The trends toward the effectiveness of the ICT-based intervention on physical and cognitive performance were analyzed by applying a single-subject research design. Additional single-subject analyses of the IG over the seven assessment times revealed more positive than negative trends. However, high intra-subject variation was found, indicating that there are other potential influences such as daily fluctuations or partly low absolute reliability of the physical performance tests among IWD. In conclusion, the InCoPE-App which enables nursing assistants to test and individually train IWD is an attractive and useful tool for nursing homes. More research is needed to further examine the feasibility and suitability of daily use of the app and find structural solutions for the implementation. Moreover, The effectiveness of the multidomain exercise intervention as delivered by nursing assistants using the InCoPE-App needs to be investigated in a larger sample of IWD.

## Data Availability

The data presented in this study are available on request from the corresponding author. The data are not publicly available due to ethical restrictions.
